# Three-Dimensional Integral Imaging with Enhanced Lateral and Longitudinal Resolutions Using Multiple Pickup Positions

**DOI:** 10.3390/s22239199

**Published:** 2022-11-26

**Authors:** Jiheon Lee, Myungjin Cho

**Affiliations:** Research Center for Hyper-Connected Convergence Technology, School of ICT, Robotics, and Mechanical Engineering, IITC, Hankyong National University, 327 Chungang-ro, Anseong 17579, Kyonggi-do, Republic of Korea

**Keywords:** axially distributed sensing, integral imaging, lateral and longitudinal resolution, synthetic aperture integral imaging, volumetric computational reconstruction

## Abstract

In this paper, we propose an enhancement of three-dimensional (3D) image visualization techniques by using different pickup plane reconstructions. In conventional 3D visualization techniques, synthetic aperture integral imaging (SAII) and volumetric computational reconstruction (VCR) can be utilized. However, due to the lack of image information and shifting pixels, it may be difficult to obtain better lateral and longitudinal resolutions of 3D images. Thus, we propose a new elemental image acquisition and computational reconstruction to improve both the lateral and longitudinal resolutions of 3D objects. To prove the feasibility of our proposed method, we present the performance metrics, such as mean squared error (MSE), peak signal-to-noise ratio (PSNR), structural similarity (SSIM), and peak-to-sidelobe ratio (PSR). Therefore, our method can improve both the lateral and longitudinal resolutions of 3D objects more than the conventional technique.

## 1. Introduction

Three-dimensional (3D) image visualization is a significant issue for many applications, such as unmanned autonomous vehicles, media content, defense, and so on [1,2,3,4,5,6,7,8]. To visualize 3D images, various methods can be utilized, such as time of flight, stereoscopic imaging, holography, and integral imaging [1,9,10,11]. By utilizing incoherent light with a general camera, integral imaging [1,9,12,13,14] can be used. It is a passive multi-perspective imaging technique that obtains depth information from 2D images, which are called elemental images. Unlike stereoscopic imaging techniques, such as anaglyphs, shutter glasses, and film-patterned retarders, integral imaging can visualize 3D images in full color, full parallax, and with continuous viewing points without special viewing devices. However, since integral imaging utilizes a lenslet array, the resolution of each elemental image is limited by the number of lenses and the resolution of the sensor. Thus, integral imaging by the lenslet array may visualize the 3D image in low resolution with a shallow depth of focus.

To solve lateral and longitudinal resolutions of 3D images in integral imaging, synthetic aperture integral imaging (SAII) [1,9,13,14,15] was reported. The elemental image acquisition obtains different perspectives of elemental images by fixing the distances between scenes and the pickup plane. It can record elemental images with the same resolution as the image sensor. After obtaining high-resolution elemental images by SAII, volumetric computational reconstruction (VCR) [1,14,16,17,18,19] can be used for 3D image generation. It can reconstruct 3D image from elemental images by shifting the pixels. It shifts each elemental image and superposes them to obtain 3D information. However, 3D images may be visualized with limited resolutions because of the fixed distance between scenes and the pickup plane.

To solve this problem, elemental images captured at different pickup positions at in-depth directions are required. Thus, in this paper, we merged SAII with axially distributed sensing (ADS) [2,20,21,22]. ADS is another 3D image visualization method that records different perspectives of elemental images by moving the image sensor along the optical axis. This feature makes ADS have fewer resolution limitations caused by the fixed distance. In the reconstruction sequence, unlike SAII, ADS utilizes the relative magnification ratio for each elemental image from different distances. However, ADS may not generate the 3D images of the center part because of a lack of perspective information. In this paper, our method uses the advantages of both SAII and ADS. In the image acquisition, we obtain elemental images by moving the sensor on the pickup plane with different positions of in-depth direction. The shifting pixel and relative magnification ratio are utilized for the 3D image reconstruction. Therefore, our methods can enhance the lateral and longitudinal resolutions of 3D images simultaneously.

This paper is organized as follows. In Section 2, we present the basic concept of integral imaging, SAII, ADS, and our proposed method. Then, in Section 3, we show the experimental results for supporting the feasibility of our proposed method with the performance metrics, such as peak signal-to-noise ratio (PSNR), structural similarity (SSIM), mean squared error (MSE), and peak-to-sidelobe ratio (PSR). Finally, we conclude with our summary in Section 4.

## 2. Three-Dimensional Integral Imaging with Multiple Pickup Positions

In this section, we present the basic concepts of integral imaging, synthetic aperture integral imaging, axially distributed sensing, and our method.

### 2.1. Integral Imaging

**Figure 1:**
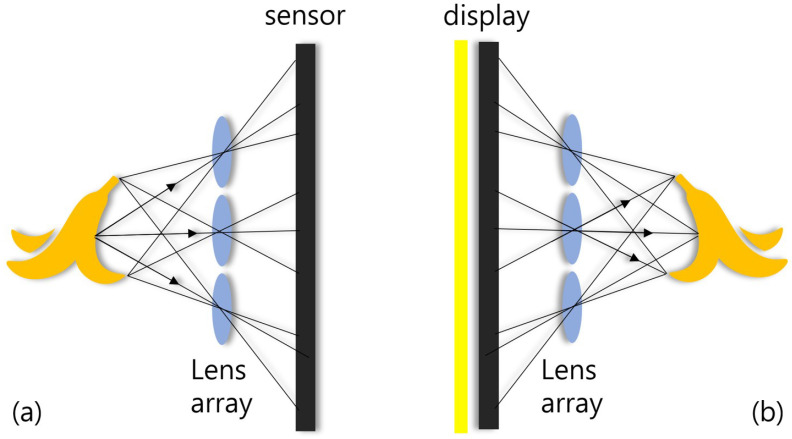
Overview of integral imaging; (**a**) is the acquisition technique and (**b**) is the reconstruction technique.

**Figure 2:**
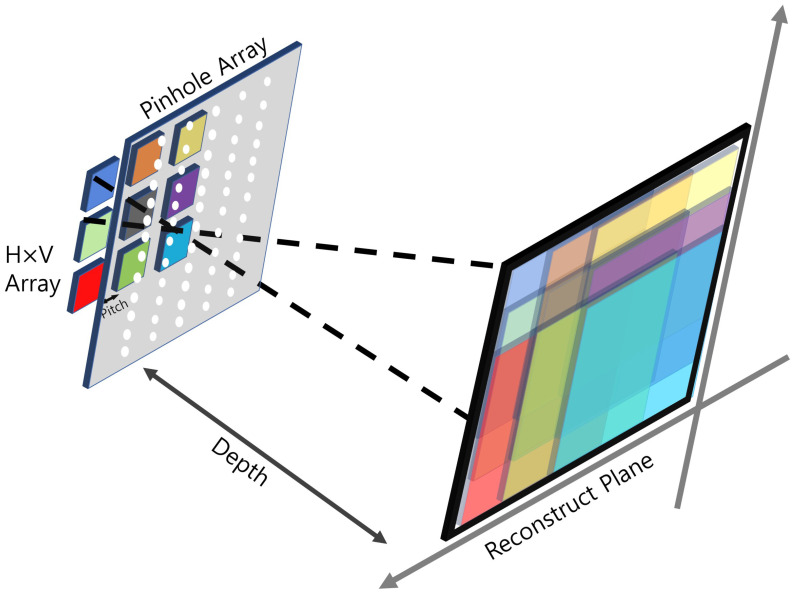
Overview of nonuniform volumetric computational reconstruction (VCR).

To visualize natural 3D scenes, integral imaging was reported by G. Lippmann in 1908 [23]. Integral imaging provides full color and full parallax 3D images with multi-perspective elemental images. Figure 1 illustrates an overview of integral imaging. As shown in Figure 1, integral imaging generates 3D images by two sequences: image acquisition and reconstruction. Image acquisition provides multi-perspective elemental images from different locations of the lenslets. In the reconstruction, there is an optical method (Figure 1b) and a computational method (Figure 2). In the optical method, the 3D image is displayed by backpropagating the elemental image through the same lenslet array. In the computational method, as shown in Figure 2, a nonuniform volumetric computational reconstruction (VCR) is utilized. It uses different shifting pixels for elemental images through various reconstruction depths and superposes them. It can be described as follows [16]

(1) $$\Delta x_{s}=\frac{N_{x}\times p_{x}\times f}{s_{x}\times z_{d}},\;\Delta y_{s}=\frac{N_{y}\times p_{y}\times f}{s_{y}\times z_{d}}$$

(2) $$\Delta x_{k}=\left\lfloor \left(k\times \Delta x_{s}\right)\right\rceil ,\;\mathrm{for}\;k=0,1,2,\cdots ,K-1$$

(3) $$\Delta y_{l}=\left\lfloor \left(l\times \Delta y_{s}\right)\right\rceil ,\;\mathrm{for}\;l=0,1,2,\cdots ,L-1$$

(4) $$I{\left(x,y,z_{d}\right)}_{VCR}=\frac{1}{O{\left(x,y,z_{d}\right)}_{VCR}}\sum_{k=0}^{K-1}\sum_{l=0}^{L-1}E_{kl}\left(x+\Delta x_{k},y+\Delta y_{l}\right)$$

where $\Delta x_{s},\Delta y_{s}$ are the actual shifting pixels in a real number, $N_{x},N_{y}$ are the number of pixels for each elemental image in x and y directions, $p_{x},p_{y}$ are the pitches for the elemental images, *f* is the focal length of the camera, $s_{x},s_{y}$ are the sensor sizes of the camera, $z_{d}$ is the reconstruction depth, $\Delta x_{k},\Delta y_{l}$ are the shifting pixels assigned to the *k*th row and *l*th column elemental images, K,L are the number of row and column elemental images, ⌊·⌉ is the round operator, $O{\left(x,y,z_{d}\right)}_{VCR}$ is the overlapping matrix for nonuniform VCR, $E_{kl}$ is the *k*th row, *l*th is the column elemental image, and $I{\left(x,y,z_{d}\right)}_{VCR}$ is the 3D image by the nonuniform VCR. However, since the resolutions of elemental images are limited by the number of lenses and the resolution of the sensor, the resolutions of 3D images may be degraded. To solve this problem, high-resolution elemental images are required in integral imaging.

### 2.2. Synthetic Aperture Integral Imaging

**Figure 3:**
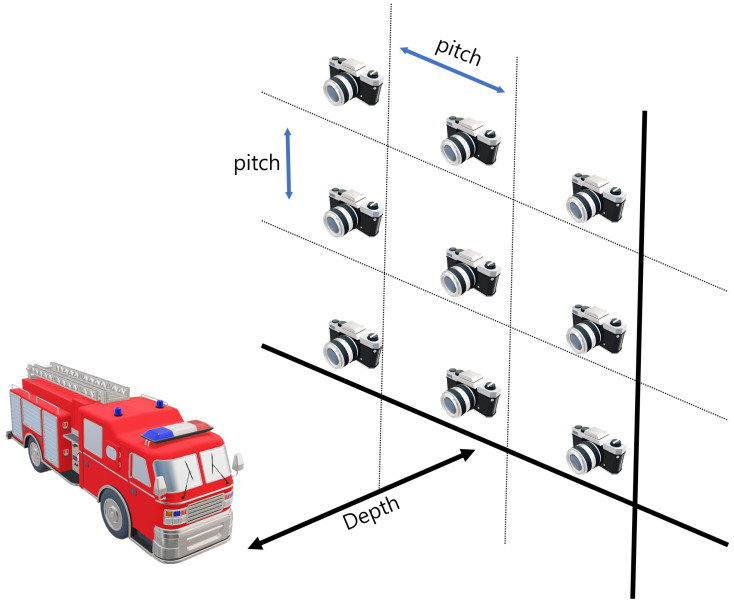
Illustrations of synthetic aperture integral imaging (SAII).

**Figure 4:**
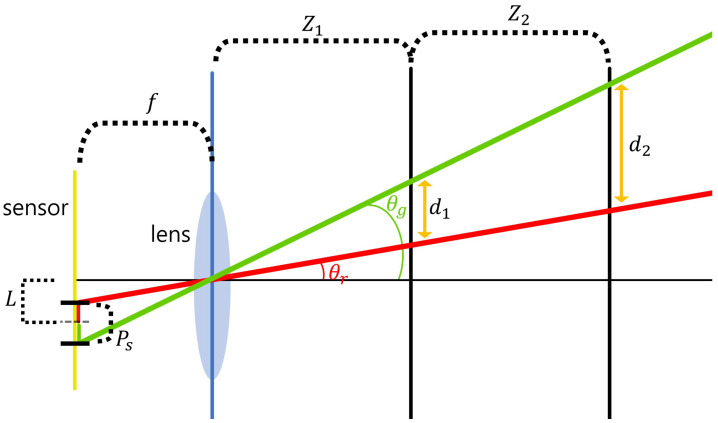
Illustration of the resolution problem.

Synthetic aperture integral imaging (SAII) is an elemental image acquisition technique. Unlike lenslet array-based integral imaging, SAII utilizes a full sensor for capturing each elemental image. Therefore, 3D images by SAII can have high resolutions. Figure 3 illustrates the SAII. As shown in Figure 3, it fixes the distance between the 3D object and the pickup plane and generates the elemental images by moving the image sensor on the pickup plane with the same pitch. However, 3D objects by using elemental images with single distances may not be visualized well due to the limited resolutions. Figure 4 illustrates the resolution problems of 3D image visualizations. In Figure 4, $P_{s}$ is the pixel size, *f* is the focal length, $Z_{n}$ is the distance between the sensor and 3D scenes, *L* is the distance between the center of the sensor and pixel, $\theta_{r},\theta_{g}$ are the angles of the red and green rays, and $d_{n}$ is the distance where we recognize a pixel as single information. It can be described as follows:

(5) $$\theta_{r}=\tan^{-1}\left(\frac{L-(\frac{P_{s}}{2})}{f}\right),\;\theta_{g}=\tan^{-1}\left(\frac{L+(\frac{P_{s}}{2})}{f}\right)$$

(6) $$d_{n}=\left(\tan\left(\theta_{g}\right)-\tan\left(\theta_{r}\right)\right)\sum_{n=1}^{N}Z_{n}$$

As shown in Figure 4 and Equations (5) and (6), the distance between the sensor and the scene ($\sum_{n=1}^{N}\left(Z_{n}\right)$) is the main factor for calculating the distances ($d_{n}$). In short, as the distance between the 3D object and the pickup plane decrease, the acquisition area per pixel becomes smaller. Therefore, it can obtain more precise information.

### 2.3. Axially Distributed Sensing

**Figure 5:**
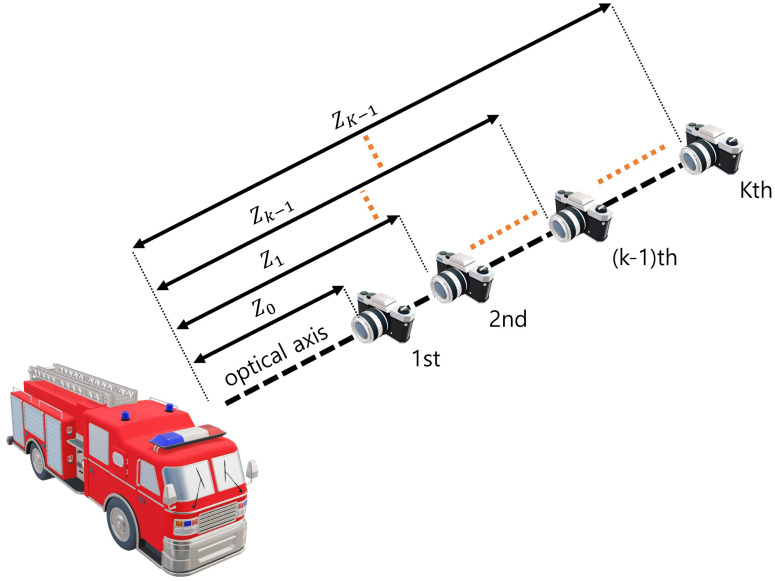
Image acquisition in axially distributed sensing (ADS).

Axially distributed sensing obtains different perspectives of the images by changing the distance between the pickup and reconstruction plane. Then, it can reconstruct the 3D image by using a relative magnification ratio for each elemental image. Figure 5 illustrates image acquisition in ADS. As shown in Figure 5, the image sensor is aligned on the optical axis with different depths. These depth differences lead to ADS having fewer resolution limitations, which are caused by the distance between the 3D object and the pickup plane. Elemental images by ADS contain different pickup areas per pixel. Therefore, in ADS reconstruction, a relative magnification ratio can be utilized. It can be described as follows [2,9,20,21]

(7) $$M_{k}=\frac{z_{k}}{z_{o}}$$

(8) $$O{\left(x,y,z_{d}\right)}_{ADS}=\sum_{k=0}^{K-1}1\left(\frac{x}{M_{k}},\frac{y}{M_{k}}\right)$$

(9) $$I{\left(x,y,z_{d}\right)}_{ADS}={\frac{1}{O(x,y,z_{d})}}_{ADS}\sum_{k=0}^{K-1}E_{k}\left(\frac{x}{M_{k}},\frac{y}{M_{k}}\right)$$

where $z_{o}$ is the nearest distance between the pickup position and the reconstruction plane, $z_{k}$ is the distance between the *k*th pickup position and the reconstruction plane, $M_{k}$ is the relative magnification ratio between $z_{k}$ and $z_{o}$, 1 is the matrix, $O{\left(x,y,z_{d}\right)}_{ADS}$ is the overlapping matrix for ADS, $E_{k}$ is the elemental image at the *k*th pickup position, $I{\left(x,y,z_{d}\right)}_{ADS}$ is the 3D image by ADS. However, since the center parts of elemental images have no perspectives, ADS may not reconstruct the center part of a 3D scene.

### 2.4. Our Method

**Figure 6:**
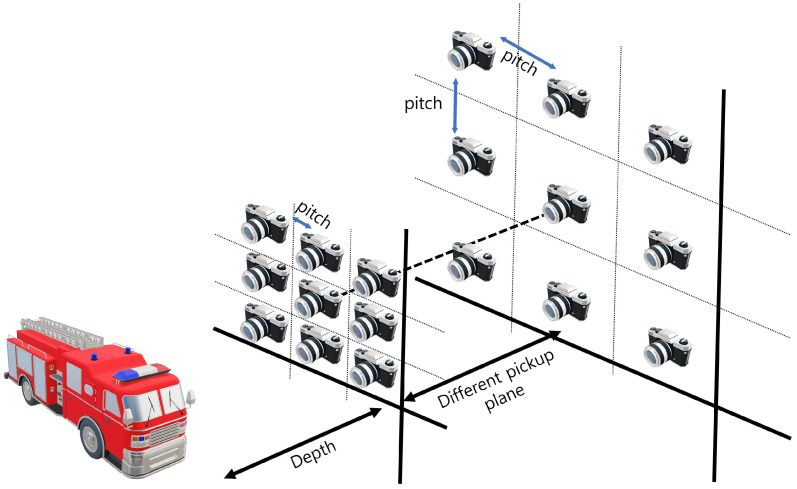
Sensor alignment in our method.

To visualize 3D images with enhanced lateral and longitudinal resolutions, we merged the SAII, ADS, and VCR techniques in this paper. In elemental image acquisition, we set the camera arrays at different pickup planes with different lateral perspectives. Figure 6 illustrates the alignment of the camera array in our method. As shown in Figure 6, elemental images with different perspectives are recorded per pickup plane. On each pickup plane, different pitches among cameras are considered. In Equation (1), the shifting pixels of each elemental image can be decided by the number of pixels ($N_{x}$), pitch ($p_{x}$), focal length (*f*), sensor size ($s_{x}$), and depth between the pickup and reconstruction plane ($z_{d}$). However, the number of pixels, focal lengths, and sensor sizes are constant values when the camera and lens are chosen. Thus, in this case, the shifting pixel can be described as follows

(10) $$\Delta x_{s}\propto k\frac{p_{x}}{z_{d}}$$

**Figure 7:**
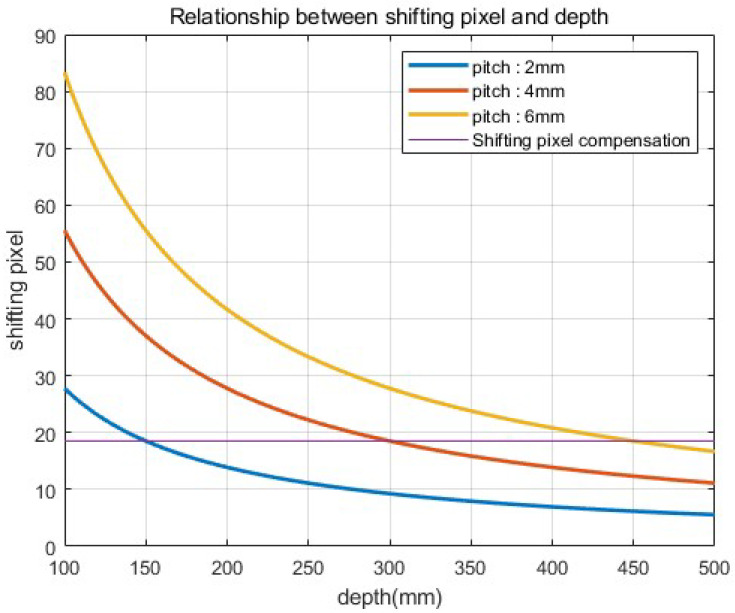
Relationship between shifting pixel and depth.

*k* is the merged constant value of $N_{x}$, $s_{x}$, and *f*. In Equation (10), shifting pixels ($\Delta x_{s}$) are considered by pitch ($p_{x}$) and depth ($z_{d}$). Figure 7 illustrates the relationship between the shifting pixel, pitch, and depth. As shown in Figure 7, the number of shifting pixels gradually decreases as the depth increases. However, in Figure 7, the shifting pixel can be compensated by using a higher pitch. Therefore, a higher pitch is used for the longest pickup plane to compensate for the lack of shifting pixels. Then we reconstruct the elemental images by considering various shifting pixels and the relative magnification ratio. It is described as follows:

(11) $$N_{x}^{m}=N_{x}^{0}\times \frac{z_{m}}{z_{0}},\;\mathrm{for}\;m=0,1,2,\cdots ,M-1$$

(12) $$N_{y}^{m}=N_{y}^{0}\times \frac{z_{m}}{z_{0}},\;\mathrm{for}\;m=0,1,2,\cdots ,M-1$$

(13) $$\Delta x_{m}^{s}=\frac{N_{x}^{m}\times f\times p^{m}}{s_{x}\times z_{d}^{m}},\;\mathrm{for}\;m=0,1,2,\cdots ,M-1$$

(14) $$\Delta y_{m}^{s}=\frac{N_{y}^{m}\times f\times p^{m}}{s_{y}\times z_{d}^{m}},\;\mathrm{for}\;m=0,1,2,\cdots ,M-1$$

(15) $$\Delta x_{km}^{s}=\left\lfloor \left(k\times x_{m}^{s}\right)\right\rceil ,\;\mathrm{for}\;k,m=0,1,2,\cdots ,K-1,M-1$$

(16) $$\Delta y_{lm}^{s}=\left\lfloor \left(l\times y_{m}^{s}\right)\right\rceil ,\;\mathrm{for}\;l,m=0,1,2,\cdots ,L-1,M-1$$

(17) $$E_{klm}=\beta \left(N_{x}^{m},N_{y}^{m}\right),\;\mathrm{for}\;k,l,m=0,1,2,\cdots ,K-1,L-1,M-1$$

(18) $$E_{klm}^{p}=\Theta \left(E_{klm},N_{x}^{M-1},N_{y}^{M-1})\right),\;\mathrm{for}\;k,l,m=0,1,2,\cdots ,K-1,L-1,M-1$$

(19) $$O_{klm}^{p}=\Theta \left(1\left(E_{klm}\right),N_{x}^{M-1},N_{y}^{M-1})\right),\;\mathrm{for}\;k,l,m=0,1,2,\cdots ,K-1,L-1,M-1$$

(20) $$O\left(x,y,z\right)=\sum_{m=0}^{M-1}\sum_{k=0}^{K-1}\sum_{l=0}^{L-1}O_{klm}^{p}\left(x+\Delta x_{km}^{s},y+\Delta y_{lm}^{s},z_{m}\right)$$

(21) $$I\left(x,y,z\right)=\frac{1}{O(x,y,z)}\sum_{m=0}^{M-1}\sum_{k=0}^{K-1}\sum_{l=0}^{L-1}E_{klm}^{p}\left(x+\Delta x_{km}^{s},y+\Delta y_{lm}^{s},z_{m}\right)$$

where $z_{0}$ is the nearest distance between the pickup and reconstruction plane, *M* is the number of pickup planes, and $z_{m}$ is the distance between the *m*th pickup and reconstruction plane. $N_{x}^{0},N_{y}^{0}$ are the number of pixels for the elemental image located at the nearest distance between the pickup and the reconstruction plane, $N_{x}^{m},N_{y}^{m}$ are the number of pixels for the elemental image located at the distance between the *m*th pickup and reconstruction plane, $\Delta x_{m}^{s},\Delta y_{m}^{s}$ are the actual shifting pixels for the *m*th pickup plane, *f* is the focal length of the camera lens, $p^{m}$ is the pitch among cameras at the *m*th pickup plane, $z_{d}^{m}$ is the depth between the *m*th pickup and the reconstruction plane, $\Delta x_{km}^{s},\Delta y_{lm}^{s}$ are the shifting pixels for the *k*th row and *l*th column elemental image on the *m*th pickup plane, $E_{klm}$ is the *k*th row, *l*th column, and *m*th pickup plane elemental image with $N_{x}^{m}\times N_{y}^{m}$ pixels, β is the magnification function that magnifies the elemental image by using the bicubic interpolation method with $N_{x}^{m}\times N_{y}^{m}$ pixels, Θ is the zero padding function, $E_{klm}^{p}$ is the expanded elemental image for reconstruction by our method, $O_{klm}^{p}$ is the one matrix with zero padding, O(x,y,z) is the overlapping matrix for the reconstruction by our method, and I(x,y,z) is the reconstructed 3D image by our method.

**Figure 8:**
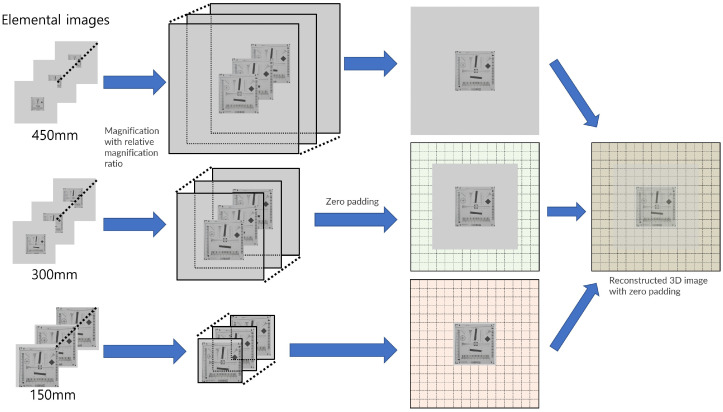
Procedure of our computational reconstruction.

Figure 8 illustrates the procedure of our computational reconstruction. In the procedure, we needed to calculate the relative magnification ratio for every elemental image and resize all elemental images considering this magnification ratio ($\frac{z_{m}}{z_{0}}$). Then, we calculated the shifting pixels ($\Delta x_{km}^{s},\Delta y_{lm}^{s}$) and assigned them to every elemental image. For convenience, in the reconstruction sequence, we applied zero padding with ($N_{x}^{M-1},N_{y}^{M-1}$) sizes to every elemental image. Finally, 3D images with enhanced lateral and longitudinal resolutions can be reconstructed.

## 3. Results of the Simulation

### 3.1. The First Simulation Setup

**Figure 9:**
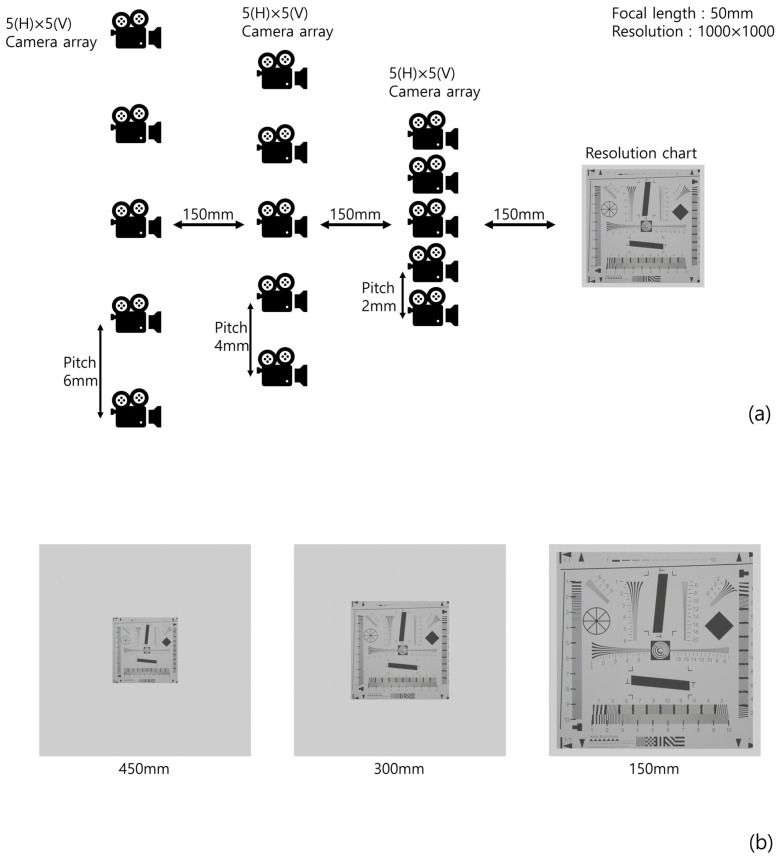
(**a**) Simulation setup and (**b**) 3D scene for each depth.

To prove the feasibility of our method, we implemented a simulation using ‘Blender’. Figure 9 illustrates the simulation setup and 3D scenes, where 5(H) × 5(V) camera arrays for each (different) depth are used, the focal length is *f* = 50 mm, the pitches of the camera arrays are 6, 4, and 2 mm, to set the same pitch for each pickup plane (Figure 7), the sensors size is 36(H) × 36(V) mm, and 1000(H) × 1000(V) is the number of pixels used for recording each elemental image. The conventional method only used the elemental images at the furthest pickup plane to reconstruct the 3D images. We used a resolution chart [24] as the object to prove the feasibility of our method.

### 3.2. The First Simulation Result

**Figure 10:**
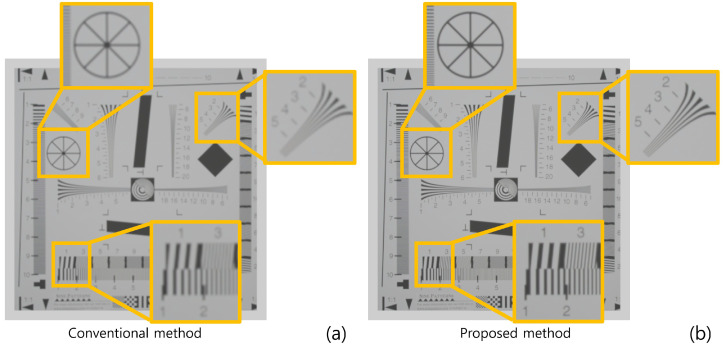
Reconstructed 3D images at 150 mm by (**a**) the conventional method and (**b**) the proposed method.

**Figure 11:**
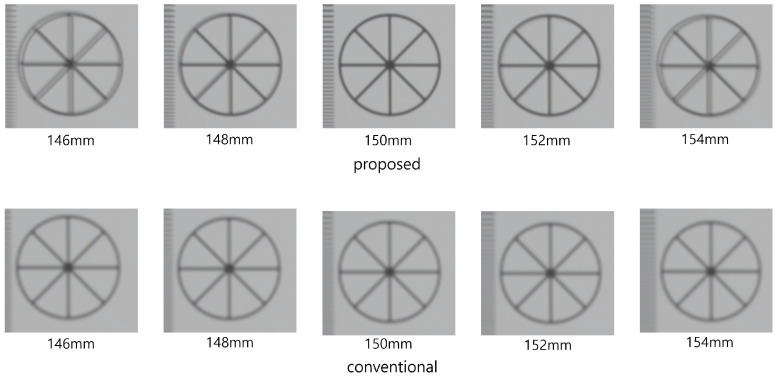
3D sample images via various depths.

Figure 10 shows the simulation results reconstructed at 150 mm. As shown in Figure 10, both conventional and proposed methods visualize 3D images correctly. However, our method has better visual quality than the conventional method. To show the feasibility of visual quality enhancement, we calculated the performance metrics, such as peak signal-to-noise ratio (PSNR), mean squared error (MSE), and structural similarity (SSIM) by using the cropped image in Figure 10. Table 1 shows the performance metric results. As shown in Table 1, sample images reconstructed by our methods have lower MSE and higher SSIM and PSNR values than the conventional method. Moreover, when we reconstruct the 3D sample images via various depths, the proposed method has better depth resolution than the conventional method. Figure 11 shows the 3D sample images reconstructed via various depths. As shown in Figure 11, it is difficult to find the correct depth (150 mm) in the conventional method. In contrast, the correct depth can be found easily in our proposed method.

### 3.3. The Second Simulation Setup

**Figure 12:**
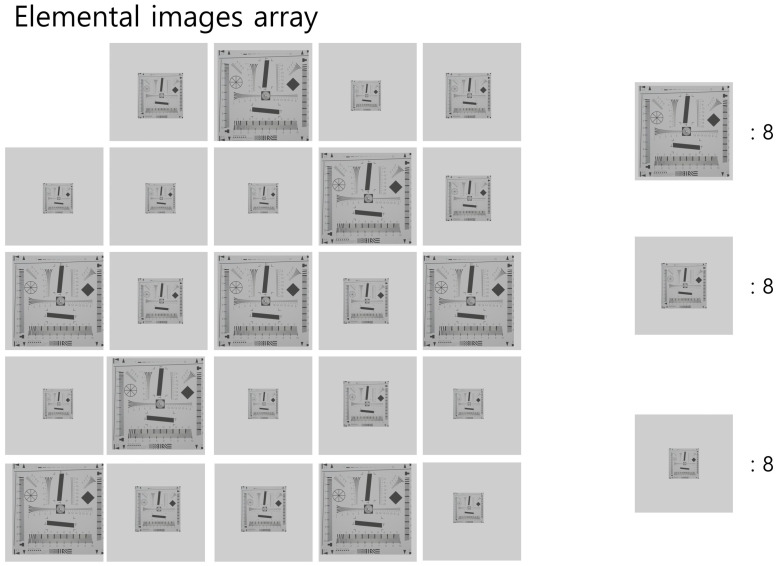
Elemental images of the second simulation.

However, the first simulation was unfair, because the proposed method used three times the elemental images than the conventional method. Thus, in the second simulation, we used fewer elemental images for the proposed method than the conventional method. Figure 12 shows the elemental images used in the second simulation. As shown in Figure 12, we extracted eight elemental images on every pickup plane.

### 3.4. Simulation Result

**Figure 13:**
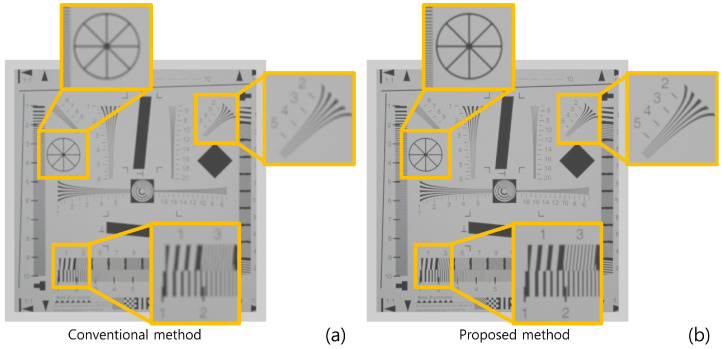
Reconstructed 3D images in the second simulation by (**a**) the conventional method and (**b**) proposed method.

**Figure 14:**
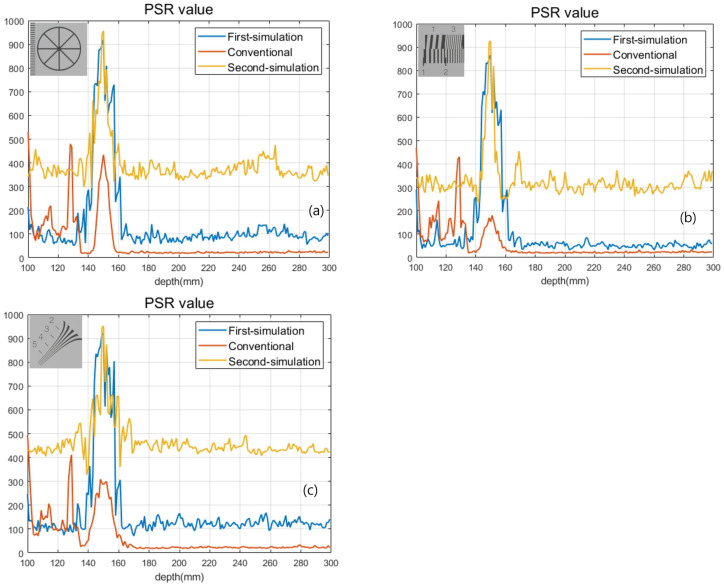
PSR values for (**a**) circle sample, (**b**) line sample, and (**c**) diagonal sample.

Figure 13 and Table 2 show the result of the second simulation. As shown in Figure 13 and Table 2, our proposed method has better visual quality than the conventional method. Finally, we calculated the peak-to-sidelobe ratio (PSR) value to show the enhancement of the depth resolution as shown in Figure 14. Each plot used a different sample (in the exact same locations as Figure 13) from the original image. As shown in Figure 14, our method provides a higher peak on the correct depth compared to the conventional method.

## 4. Conclusions

In this paper, we propose a 3D visualization method with an enhanced 3D resolution by merging SAII, VCR, and ADS. It may improve the lateral and longitudinal resolutions of 3D images by acquiring elemental images from different depths. Considering PSNR, MSE, SSIM, and PSR values as performance metrics, we prove that our method has better results than the conventional method. We believe that our method can be applied to many industries, such as unmanned autonomous vehicles, media content, and defense. However, it has some drawbacks. First, it requires great effort to implement real optical experiments due to lens aberration and alignment problems. Thus, we need more research and accurate optical components to apply our method in real optical experiments. Second, its processing speed is slower than the conventional method because it requires more processing steps to use the relative magnification ratio. Therefore, we will investigate the solutions to these drawbacks in future work.

## Figures and Tables

**Table 1:** Performance metrics of the first simulation results.

Image	Method	MSE	PSNR	SSIM
Line	Proposed method	**141.175**	**26.6167**	**0.912548**
	Conventional	412.612	21.9755	0.735038
Diagonal line	Proposed method	**70.2242**	**29.6659**	**0.934659**
	Conventional	191.215	25.3156	0.822554
Circle	Proposed method	**120.501**	**27.3209**	**0.928587**
	Conventional	362.636	22.5361	0.775436

**Table 2:** Performance metrics of the second simulation results.

Image	Method	MSE	PSNR	SSIM
Line	Proposed method	**150.162**	**26.3652**	**0.907574**
	Conventional	412.612	21.9755	0.735038
Diagonal line	Proposed method	**71.6068**	**29.5813**	**0.934551**
	Conventional	191.215	25.3156	0.822554
Circle	Proposed method	**128.099**	**27.0554**	**0.925271**
	Conventional	362.636	22.5361	0.775436

## Data Availability

Not applicable.
